# Social brains on drugs: tools for neuromodulation in social neuroscience

**DOI:** 10.1093/scan/nst113

**Published:** 2013-07-24

**Authors:** Molly J. Crockett, Ernst Fehr

**Affiliations:** ^1^Department of Economics, Laboratory for Social and Neural Systems Research, University of Zurich, Zurich 8006, Switzerland and ^2^Wellcome Trust Centre for Neuroimaging, University College London, London WC1N 3BG, UK

**Keywords:** neuromodulation, hormones, serotonin, oxytocin, testosterone, dopamine

## Abstract

Neuromodulators such as serotonin, oxytocin and testosterone play an important role in social behavior. Studies examining the effects of these neuromodulators and others on social cognition and behavior (and their neural underpinnings) are becoming increasingly common. Here, we provide an overview of methodological considerations for those wishing to evaluate or conduct empirical studies of neuromodulation in social neuroscience.

## INTRODUCTION

One of social psychology’s most important contributions is the notion that situations are powerful determinants of human behavior (Ross *et al.*, 1991). Methodological advances in social and affective neuroscience are beginning to provide us with tools for discovering ‘how’*.* Brains are sensitive to the surrounding environment, and one mechanism through which environments shape brains is by influencing the function of neuromodulators*—*chemicals that modify neuronal dynamics, excitability and synaptic function. Neuromodulators include neurotransmitters (e.g. serotonin, noradrenaline, acetylcholine and dopamine) as well as hormones (e.g. testosterone, oxytocin and vasopressin). These chemicals may serve to prepare organisms to interact optimally with the environment, shaping behavior to fit the current context in an adaptive manner. Activation of one or more of these chemical systems is an efficient way to alter the computational properties of neural networks at a global level (Robbins and Arnsten, 2009).

Recent work has begun to examine how manipulating neuromodulators influences social cognitions and behaviors such as trust (Kosfeld *et al.*, 2005), punishment (Crockett *et al.*, 2008, 2013), moral judgment (Crockett *et al.*, 2010a), conformity (Campbell-Meiklejohn *et al.*, 2012; Stallen *et al.*, 2012) and empathy (Hurlemann *et al.*, 2010). The effects of specific neuromodulators on social behavior have been reviewed elsewhere (Insel, 2010; Eisenegger *et al.*, 2011; Crockett and Fehr, 2013; Siegel and Crockett, 2013). Here, we provide a primer for conducting and evaluating empirical studies with neuromodulatory tools, highlighting methodological issues that are particularly salient in the context of studying social behavior. This topic is important for advancing social, cognitive and affective neuroscience for at least three reasons. First, animal research provides strong evidence that neuromodulators play a crucial role in a range of important social behaviors, including affiliation, aggression and social dominance (Insel, 2010); neurobiological models of human social behavior will be incomplete without a detailed understanding of neuromodulator effects. Second, the pharmacological techniques used to study neuromodulator function in humans often produce subjective effects on mood and cognitive factors like attention and executive control. As mood, attention and executive control can exert independent influences on social behavior (Lieberman, 2003; Strack and Deutsch, 2004), designing experiments to identify ‘selective’ effects of neuromodulators on social behavior requires care and consideration. Finally, psychological disorders are often characterized by dysfunctional social cognition as well as abnormal neuromodulator function (Kishida *et al.*, 2010); research examining how neuromodulators influence healthy social cognition may pave the way for pharmacological therapies to ameliorate social disturbances in psychological disorders.

## METHODS FOR MANIPULATING NEUROMODULATORS

### Direct administration

Direct oral or intravenous administration of neuromodulators (e.g. serotonin, norepinephrine and dopamine) is not generally possible, because most of these molecules cannot cross the semi-permeable separation that prevents materials in the bloodstream from entering the brain (called the ‘blood–brain barrier’). For some neuropeptides (e.g. oxytocin and vasopressin), it may be possible to administer the compounds through the nasal passages, which bypass the blood–brain barrier; the majority of studies examining how oxytocin affects social behavior have used intranasal administration (Veening and Olivier, 2013). However, it remains unclear how intranasally administered neuromodulators enter the brain and reach the appropriate receptor sites (Churchland and Winkielman, 2012; Veening and Olivier, 2013). The hormones testosterone and estradiol, which do cross the blood–brain barrier, can be administered orally (Bos *et al.,* 2011).

### Precursor manipulation

Neuromodulator levels can be influenced by manipulating their chemical precursors, which can be amino acids or other molecules that are able to cross the blood–brain barrier. Neuromodulator production can sometimes be enhanced by increasing the availability of precursor via pharmacological or dietary supplementation or impaired by decreasing the availability of precursor via dietary depletion.

Dietary depletion of precursor results in a reversible, partial global reduction in brain neurotransmitter levels. In the precursor depletion procedure, subjects ingest an amino acid load (usually in liquid or pill form) that does not contain the precursor amino acid but does include other large neutral amino acids (LNAAs). The influx of amino acids lowers the ratio of precursor to other LNAAs. As the precursor competes with other LNAAs to enter the brain through the blood–brain barrier, lowering the precursor:LNAA ratio almost completely halts precursor transport into the brain (Booij *et al.*, 2003).

There are two techniques for dietary enhancement of neuromodulator precursors. The first, called ‘supplementation’*,* involves administering a smaller dose of the precursor over several days or weeks. The second, called ‘loading’, involves administering a large acute dose of the precursor. Supplementation and loading are able to enhance neuromodulator production when the enzyme that produces the neuromodulator is not normally saturated. For instance, serotonin production can be enhanced by supplementation or loading of its precursor, the amino acid tryptophan. This is because the rate-limiting enzyme that converts tryptophan to serotonin, tryptophan hydroxylase, is not normally saturated (Silber and Schmitt, 2010).

Further examples of precursor manipulation include tryptophan depletion (impairs serotonin production), tyrosine depletion (impairs noradrenaline and dopamine production), and L-DOPA administration (enhances dopamine production).

### Receptor agonists and antagonists

Neuromodulators work by binding to different kinds of ‘receptors’. There are many different types of receptors for each neuromodulator system, and different receptor types can have different effects on neuronal function when activated. For example, dopamine D_1_ and D_2_ receptors can have opposing effects on long-term potentiation and neuronal excitability [reviewed by Frank (2005)]. The distribution of different receptor types can vary across the brain; so for instance, D_1_ and D_2_ receptors are found in roughly equal proportions in the striatum, whereas D_1_ receptors outnumber D_2_ receptors in much of the prefrontal cortex (Hall *et al.*, 1994). The consequence of this neuronal architecture is that neuromodulators, when released, can have different effects in different brain regions according to the type of receptor activated. Some pharmacological agents directly stimulate or block neuromodulator receptors. These agents can be highly selective (targeting only a specific receptor subtype) or less so (targeting a general class of receptors and binding to multiple receptor subtypes). ‘Antagonists’ bind to the receptor and block the actions of the endogenous neuromodulator, thus impairing neuromodulator function. ‘Agonists’ bind to the receptor and mimic the actions of the endogenous neuromodulator. When agonists bind to post-synaptic receptors, their net effect is to increase neuromodulator function. However, agonists and antagonists can also influence neuromodulator function by binding to special receptors called ‘autoreceptors’. Autoreceptors are located on the neurons that produce and release neurotransmitters. When activated, autoreceptors inhibit synthesis and release of neurotransmitters. This is a negative feedback mechanism designed to keep neurotransmitter release in homeostatic balance. Meanwhile, antagonism of autoreceptors can stimulate neurotransmitter synthesis and release by blocking negative feedback brought on by endogenous neurotransmitter. Thus, when they bind to autoreceptors, agonists have the net effect of decreasing neuromodulator function, whereas antagonists have the net effect of increasing neuromodulator function. The effects of agonists and antagonists on neuromodulator function therefore depend on whether they activate presynaptic or postsynaptic receptors. Examples of such drugs include haloperidol (antagonist for multiple dopamine receptors), sulpiride (antagonist for dopamine D_2_ receptors), pramipexole (agonist for dopamine D_2_ receptors), bromocriptine (agonist for dopamine D_1_ and D_2_ receptors) and propranolol (antagonist for noradrenaline beta receptors).

### Re-uptake inhibition

Selective re-uptake inhibitors increase the concentration of neuromodulator in the synapse by blocking its presynaptic re-uptake. Re-uptake inhibitors work by blocking the presynaptic active transport mechanism in the transporter protein, located on the cell membrane that is responsible for taking up neurotransmitter from the synapse after its release. Consequently, the action of the neuromodulator on postsynaptic receptors is prolonged. Examples of re-uptake inhibitors include citalopram, paroxetine and fluoxetine (selective serotonin re-uptake inhibitors, or SSRIs); atomoxetine and reboxetine (selective noradrenaline re-uptake inhibitors, or SNRIs) and methylphenidate (a dopamine re-uptake inhibitor).

There is some evidence that acute administration of re-uptake inhibitors can under certain conditions lead to a net decrease in the release of neuromodulator. This is thought to be caused by the down-regulating effects of presynaptic autoreceptor activation. For instance, a recent study showed that a 10 mg intravenous dose of citalopram led to a net decrease in endogenous serotonin release by the raphé nuclei, brought on by enhanced serotonergic transmission within the raphé nuclei (Selvaraj *et al.,* 2012). Studies in animals suggest that the dosage used is likely to influence whether acute SSRI administration enhances or reduces 5-HT neurotransmission, with lower doses reducing 5-HT neurotransmission (via autoreceptor negative feedback) and higher doses enhancing 5-HT neurotransmission (Bari *et al.*, 2010). However, further research is needed to specify the effects of re-uptake inhibitor dosages on neurotransmission in human subjects.

### Metabolic enzyme inhibitors

The synaptic actions of neurotransmitters can be prolonged by pharmacologically restraining the metabolic enzymes that break down neurotransmitters after they are released. One example is galantamine, which inhibits the enzyme that degrades acetylcholine, thus prolonging cholinergic actions in the brain.

## PRACTICAL ISSUES IN BEHAVIORAL PSYCHOPHARMACOLOGY

### Placebo and blinding issues

One advantage of using pharmacological manipulations to study the neurobiology of social behavior is that such manipulations can establish ‘causal’ mechanisms, as long as the experiment is properly designed. Perhaps the most important feature of pharmacological experiment is the ‘double-blind placebo control’. In the experimental condition, participants receive the pharmacological agent; in the control condition, participants receive an inactive placebo. All aspects of the experimental procedure are identical aside from the administration of drug *vs* placebo. Critically, neither the experimenter nor the participants know whether they have received drug or placebo. On the experimenter side, this is important so that the experimenter does not bias the data collection process, either consciously or unconsciously. On the participant side, this is important because beliefs about whether one has received drug or placebo can influence behavior independently from the effects of the drug itself (Eisenegger *et al.*, 2009).

Maintaining double-blind conditions can be difficult, however, when the pharmacological agent induces physical side-effects such as nausea, increased heart rate or dizziness, all of which are common symptoms of drugs typically used to manipulate neuromodulators, even at relatively low doses. Note that side-effects can be more severe in a neuroimaging environment. In addition to potentially interfering with task performance and producing subjective mood effects that could independently affect the dependent measures of interest, side-effects also make it more likely that subjects will be able to distinguish between the drug and placebo.

One approach to this issue is to employ a ‘positive control’—a second pharmacological agent used as a comparison condition for the drug of interest that has a similar side-effect profile. For example, if one is interested in studying how serotonin influences social behavior, one could compare the effects of citalopram (a serotonin re-uptake inhibitor) with those of atomoxetine (a noradrenaline re-uptake inhibitor with a similar side-effect profile to citalopram) as well as placebo (Crockett *et al.*, 2010a). With this procedure, even if participants can distinguish between drug and placebo due to physical side-effects, as long as they cannot distinguish between the experimental treatment (e.g. citalopram) and the positive control (e.g. atomoxetine), some degree of blindness can be maintained. Using a positive control has the additional benefit of probing for the neurochemical selectivity of the effect of interest in terms of the neuromodulator systems involved in the process under examination.

### Controlling for beliefs

Even when one goes to great lengths to set up a double-blind placebo-controlled procedure, participants may nevertheless form beliefs about which treatment they received that can significantly affect their behavior. It is, therefore, important to ask participants to report, at the end of the experiment, their subjective beliefs about which treatment they received. This belief data can be important: a notable example comes from a recent study examining the effects of testosterone on bargaining behavior (Eisenegger *et al.*, 2009). While testosterone caused participants to make more generous offers during a bargaining game, those subjects who believed they had received testosterone (as reported in the post-experiment questionnaire) made ‘less’ generous offers, regardless of whether they actually received testosterone or placebo. The authors hypothesized that this belief effect reflects folk wisdom about testosterone: namely, that it causes antisocial or aggressive behavior. Thus, participants who believed they received testosterone may have felt ‘morally licensed’ to make less generous offers. This finding underscores the importance of measuring beliefs in these kinds of experiments, particularly when studying complex social interactions where beliefs can play a decisive role.

### Between-subjects *vs* within-subjects designs

In pharmacological studies, the drug treatment can be carried out either ‘between subjects’ (in which one group of participants receives the pharmacological agent and another matched group of participants receives placebo) or ‘within subjects’ (in which participants take part in the experiment in multiple sessions, receiving placebo in one session and the drugs in the other sessions, with the order of treatments counterbalanced across participants). Each approach has advantages and disadvantages. Within-subjects designs tend to be more powerful statistically, because each participant serves as her own comparison, error variance associated with individual differences is reduced. This is particularly important in pharmacological experiments, because there are several known genetic polymorphisms that influence the signaling properties within neuromodulator systems (e.g. the function of specific types of neuromodulator receptors). These polymorphisms could create potentially large variations between individuals in terms of their physiological response to pharmacological treatment.

Within-subjects designs are less desirable when the behavior under study is susceptible to learning/practice effects or change across time, because subjects participate in the experiment multiple times. For example, Wood *et al.* (2006) used a within-subjects design to examine the effects of tryptophan depletion on behavior in a repeated prisoner’s dilemma, in which two players learn about each other’s propensity to cooperate or defect. Tryptophan depletion reduced cooperative behavior, but only in the first experimental session, that is, when participants were naïve to the prisoner’s dilemma task and early in the process of learning about the strategy of the other player. In the second experimental session, (after subjects had already learned the other player’s strategy), tryptophan depletion had little effect (Wood *et al.*, 2006).

In addition, some social psychological paradigms are difficult (if not impossible) to conduct in a repeated-measures setting. In particular, those paradigms that involve deception pose a challenge for repeated-measures designs. Generally, when the research paradigm requires convincing subjects of something that is not true (e.g. subjects are led to believe that they are interacting with a real person, when in fact they are interacting with a computer program), it is advisable to collect self-report measures at the end of the study to assess whether the subject believed the experimenter’s cover story. However, in a repeated-measures design, collecting self-report measures of belief in the cover story at the end of the first experimental session may contaminate behavior in the second experimental session, if the self-report measures raise suspicions about the veracity of the cover story where none were present before. To avoid this possibility, one might only collect belief measures at the end of the second session; however, this approach rests somewhat on the assumption that subjects’ beliefs about the veracity of the cover story are consistent across sessions and treatments, which may not be the case (see below, section ‘Demonstrating behavioral selectivity’).

If the aim of the experiment is to examine neuromodulator effects on learning or one-shot decisions or in paradigms where within-subjects treatments are infeasible, a between-subjects design may be more appropriate. When using a between-subjects design, it is critical to ensure that the experimental group and the placebo group are matched on important characteristics such as sex, age, education and perhaps also personality traits and genetic polymorphisms relevant to the neuromodulator system under study. Although a detailed review of the effects of genetic polymorphisms is beyond the scope of this review, it is worth mentioning that the effects of pharmacological manipulations can vary according to genotype (Eisenegger *et al.*, 2010; Rogers, 2010), an issue worth considering when designing pharmacological experiments, especially those with between-subjects designs.

### Timing of drug administration

The time course of the effects of pharmacological manipulations varies depending on the agent used and the method of administration. Following oral administration of drugs, peak concentrations tend to occur within a few hours, while intravenous and intranasal administrations tend to have faster-acting effects. Meanwhile, dietary depletions take considerably longer to exert their effects, on the order of 5–6 h. It is important to precisely time the experimental procedure such that the dependent measures are collected at the time point most likely to coincide with peak drug effects.

If more than one pharmacological agent is used and the drugs have different time courses, a multiple placebo procedure can be employed to maintain double-blinded conditions. For example, consider a study comparing the effects of levodopa and citalopram with placebo, where levodopa reaches peak concentration 1 h after administration and citalopram reaches peak concentration 3 h after administration. The levodopa group receives levodopa 1 h prior to testing and a placebo pill 3 h prior to testing. The citalopram group receives placebo 1 h prior to testing and citalopram 3 h prior to testing. Finally, the placebo group receives placebo at both 1 and 3 h prior to testing. Thus, across conditions all subjects receive treatment at both 1 h and 3 h pre-testing, but neither the subjects nor the experimenters know the contents of the treatment, maintaining double-blinded conditions.

Another consideration related to timing relates to experiments using within-subjects designs. Drugs differ in the amount of time they take to leave the body. In within-subjects designs, it is important that testing sessions are spaced sufficiently far apart for a full washout to occur, generally at least 1 week. When recruiting subjects, it is also worth checking whether they have recently participated in other studies involving pharmacological manipulations. In addition, as other substances such as alcohol, caffeine and recreational drugs can have prolonged effects in the brain and can interact with your experimental treatment, it is important to make sure subjects abstain from these substances for at least 24 h prior to participation and throughout the duration of the study (for within-subjects designs).

Finally, if females are included in the study, it is worth considering whether to control for menstrual phase cycle, because endogenous sex hormones could potentially interact with the neuromodulator under study. If this is a concern, it is good practice to restrict female participants to those with a regular menstrual cycle who are not taking oral contraceptives and to test them in the early follicular phase of the cycle, when the endogenous level of sex hormones tends to be low and stable.

### Choosing the appropriate dose

The chosen dose of the drug can have important implications for the effects of the manipulation. For example, low doses of sulpiride (a D2 antagonist; e.g. 100–200 mg) are thought to primarily exert effects on presynaptic receptors, potentially leading to a net stimulatory effect on DA neurotransmission, whereas higher doses (e.g. 400–800 mg) are more likely to act postsynaptically and reduce DA actions on D2 receptors (Di Giovanni *et al.*, 1998). Meanwhile, low doses of SSRIs (e.g. 10 mg) can reduce serotonin release by enhancing the actions of endogenous serotonin on presynaptic autoreceptors (Selvaraj *et al.*, 2012), whereas higher doses (e.g. ≥30 mg) may be sufficient to enhance serotonin neurotransmission in terminal regions. In line with this idea, studies in animals have shown that different doses of SSRIs have different effects on motivated behavior (Bari *et al.*, 2010). In humans, the effects of pharmacological manipulations at the molecular level are incompletely understood and should be interpreted with caution. Future studies combining pharmacological manipulations with positron emission tomography (PET) are needed to elucidate the effects of these manipulations on endogenous neurotransmitter synthesis and release.

### Blood plasma measures

As noted previously, there are widespread individual differences in physiological responses to pharmacological treatments. Collecting additional data from blood samples can provide information about the nature of these individual differences and how they interact with the treatment.

When conducting precursor depletion or supplementation studies, it is essential to collect blood samples both at baseline (i.e. before subjects ingest the amino acids) and just before testing. This enables confirmation that plasma levels of precursor, and the ratio of precursor to LNAAs, were indeed depleted by the manipulation (Booij *et al.*, 2003), because the procedure can be compromised by participant non-compliance (e.g. if the participant consumes any foods containing the precursor during the waiting period or fails to comply with the supplementation regime). Individual differences in plasma precursor levels can also serve as covariates in behavioral and neuroimaging analyses. For instance, individual differences in plasma tryptophan:LNAA ratios predicted individual differences in the effects of tryptophan depletion on impulsive choice behavior (Crockett *et al.*, 2010b) and subject-specific plasma tryptophan:LNAA ratios influenced reward prediction error responses in the putamen (Seymour *et al.*, 2012).

Unlike precursor depletion and supplementation studies, drug administration studies do not necessarily require measurement of plasma levels of the drug, as these procedures are less vulnerable to participant non-compliance. However, it can still be useful to collect blood samples to measure plasma levels of the drug, which sometimes co-vary with the drug’s behavioral and/or neural effects. For example, Chamberlain *et al.* (2009) found that plasma levels of atomoxetine predicted right inferior frontal gyrus activity during response inhibition (Chamberlain *et al.*, 2009).

Note that for substances that cross the blood–brain barrier (e.g. tryptophan or atomoxetine), plasma levels of the substance are likely correlated with brain levels of that substance. However, for substances that have low penetration of the blood–brain barrier (e.g. oxytocin or vasopressin), plasma levels are not necessarily indicative of brain levels of that substance. Studies that use plasma levels of a substance with weak penetration of the blood–brain barrier to make claims about brain levels of that substance should therefore be interpreted with caution (Churchland and Winkielman, 2012).

### Controlling for subjective experience

As pharmacological manipulations can have physical side-effects or influence mood more generally, it is important to rule out these factors as causal mediating forces in the effects of neuromodulators on social behavior. Subjective rating scales are a useful tool for assessing these effects. Commonly used scales include the Visual Analogue Scales (Bond and Lader, 1974) and the Positive and Negative Affect Scales (Watson *et al.*, 1988). These scales assess the effects of the pharmacological manipulation on subjective feelings such as alertness, calmness, irritability, contentedness, drowsiness, anxiety, nausea, dizziness and positive and negative affect. Drug-induced changes in physical side-effects or mood can be included as regressors of no interest in statistical models capturing the effects of pharmacological manipulations on social behavior.

### Demonstrating behavioral selectivity

It is relatively straightforward to pick some behavior *Z* and perform a pharmacological study to examine the effects of neuromodulator *X* on behavior *Z.* However, to make the claim that *X* has a *selective effect* on *Z* requires some methodological sophistication. As social behaviors are complex constructs incorporating several more basic perceptual and motivational processes (many of which may be sensitive to the neuromodulator in question), to make claims about neuromodulators’ behavioral selectivity, one must control for these basic processes where possible.

An example of this comes from a study on how oxytocin affects behavior in a game of trust. In this study, oxytocin increased subjects’ trusting behavior by 17%, relative to a placebo control group (Kosfeld *et al.*, 2005). But before the authors could conclude that oxytocin modulates trust specifically, they had to rule out the possibility that oxytocin simply altered sensitivity to risk, as trust involves a degree of risk taking. To do this, they conducted a risk experiment, in which subjects faced exactly the same decisions as in the trust game, but removed from a social context: the interaction partner was replaced with a computer. Critically, oxytocin did ‘not’ affect behavior in the risk experiment, indicating that the effects of oxytocin on trust are specific to the social context.

Another issue worth considering is the possibility that neuromodulators may influence susceptibility to deception and/or experimenter demand effects. Oxytocin, for example, enhances trust in some settings (Van IJzendoorn and Bakermans-Kranenburg, 2012) and there is no a priori reason to assume that these effects do not extend to trust in the experimenter. Thus, paradigms in which experimenter demand effects are expected to be high, and/or those involving deception of subjects by the experimenters, may show an effect of oxytocin on the behavior of interest not because oxytocin actually influences the behavior of interest, but because it enhances trust in the experimenter and consequently, subjects’ engagement with the task. It is therefore critical to collect, where possible, independent measures of subjects’ beliefs about the veracity of the experimental set-up, engagement with the task and desire to please the experimenter, in order to control for possible neuromodulator effects on these measures.

## CONCLUSION

One important challenge for human psychopharmacology is the scarcity of methods for assessing the molecular-level effects of pharmacological manipulations *in vivo.* Although it is straightforward to investigate how drug treatments alter behavior and brain hemodynamic responses, these measures reflect downstream effects of the changes in neurotransmission at the molecular level. Previous pharmacological studies in humans provide evidence of behavioral effects, but can say very little about the underlying changes in neurotransmission. PET imaging can provide quantitative measurements of endogenous neurotransmitter release (Martinez *et al.*, 2003; Selvaraj *et al.*, 2012); future studies could combine pharmacological manipulations with PET, functional magnetic resonance imaging and behavioral measurements to link the drug treatment to changes in endogenous neurotransmitter release to changes in neural activity to changes in behavior.

As social neuroscience progresses, it will become ever more important to employ methods that enable inferences about cause and effect. The combination of pharmacological manipulations with neuroimaging will facilitate the identification of the brain networks that are causally involved in generating social cognition and behavior. These kinds of studies will bring us closer to a mechanistic understanding of social interaction.

## Conflict of Interest

None declared.
